# Situational Judgment Tests as a method for measuring personality: Development and validity evidence for a test of Dependability

**DOI:** 10.1371/journal.pone.0211884

**Published:** 2019-02-27

**Authors:** Gabriel Olaru, Jeremy Burrus, Carolyn MacCann, Franklin M. Zaromb, Oliver Wilhelm, Richard D. Roberts

**Affiliations:** 1 University of Kassel, Kassel, Germany; 2 American College Testing, Iowa City, Iowa, United States of America; 3 University of Sydney, Sydney, Australia; 4 National Authority for Measurement and Evaluation in Education, Ramat Gan, Israel; 5 Ulm University, Ulm, Germany; 6 Rad Science Solution, Philadelphia, Pennsylvania, United States of America; Leibniz Institute for Educational Trajectories, GERMANY

## Abstract

Situational Judgment Tests (SJTs) are criterion valid low fidelity measures that have gained much popularity as predictors of job performance. A broad variety of SJTs have been studied, but SJTs measuring personality are still rare. Personality traits such as Conscientiousness are valid predictors of many educational, work and life-related outcomes and SJTs are less prone to faking than classical self-report measurements. We developed an SJT measure of Dependability, a core facet of Conscientiousness, by gathering critical incidents in semi-structured interviews using the construct definition of Dependability as a prompt. We examined the psychometric properties of the newly developed SJTs across two studies (N = 546 general population; N = 440 sales professionals). The internal validity of the SJTs was examined by correlating the SJT scores with related self-report measures of Dependability and Conscientiousness, as well as testing the unidimensionality of the measure with CFA. Additionally, we specified a bi-factor model of SJT, self-report and behavioral checklist measures of Dependability accounting for common and specific measurement variance. External validity was examined by correlating the SJT scale and specific factor with work-related outcomes. The results show that the Dependability SJTs with an expert based scoring procedure were psychometrically sound and correlated moderately to highly with traditional self-report measures of Dependability and Conscientiousness. However, a large proportion of SJT variance cannot be accounted for by personality alone. This supports the notion that SJTs measure general domain knowledge about the effectiveness of personality-related behaviors. We conclude that SJT measures of personality can be a promising addition to classical self-report assessments and can be used in a wide variety of applications beyond measurement and selection, for instance as formative assessments of personality.

## Introduction

Situational Judgment Tests (SJTs) are low fidelity simulations that in recent decades have been widely adopted in the workforce for personnel selection [1]. SJTs typically present a situation describing a dilemma or problem along with different response options which test-takers evaluate using their knowledge, skills, abilities, and/or other characteristics (2). Indeed, numerous studies have demonstrated SJTs to be efficient–that is cheap and easy to create, administer and evaluate–and criterion-valid predictors of many work-related outcomes, such as job performance, interpersonal skills, or leadership (M_ρ_ = .20-.30) [2,3]. As a result, it has become very common in the workforce for employers to incorporate SJTs as one of their tools for personnel selection [1].

Although SJTs are already established as criterion-valid predictors of work-related outcomes [3–5], there is little consensus on what SJTs actually measure [6]. In addition to the original interpretation of SJTs as measures of tacit or job knowledge [7,8], SJTs have also been understood as predictive methods without a clear internal structure [9], as measures of situation-specific reactions [10] (but also see [11]), or as measures of dimensions, such as personality [2]. Jackson and colleagues [6] evaluated these perspectives by using variance decomposition [12] to identify relevant aspects captured with SJTs. Their results suggest that situations explain little variance in the SJT responses (i.e., around 1–3%) [12], as do domains (i.e., 0–6%). Instead, they found that the majority of SJT variance can be attributed to ability differences between respondents (i.e., 48–67%), which might be in line with the original definition of SJTs as measures of knowledge. However, the SJTs evaluated by Jackson and colleagues [6] were used as selection tools for job applicants, and were thus developed primarily with the intent of maximizing predictive validity. Christian and colleagues [2] suggest that SJTs can, and should, be developed with the goal of measuring specific constructs, which would arguably increase the trait variance captured by this assessment method. Newer studies that follow this approach have shown the potential of SJT measures of personality [13,14]. In this article, contribute to the ongoing discussion by developing SJT measures of personality (i.e., Dependability) and examine the construct validity of the newly developed measures.

### SJT versus traditional self-report measures of personality

A reasonable question to ask at this point is how personality SJTs can contribute to research and practice, compared to self-report measures of personality or traditional SJTs. Personality traits, such as Conscientiousness, Emotional Stability, and Agreeableness, are well established predictors of many relevant life outcomes (e.g., life satisfaction, longevity) [15,16], as well as academic [17] and work-related performance [18]. For example, in education, a meta-analysis on the relations between cognitive ability and personality with academic outcomes has shown that in secondary and tertiary education, Conscientiousness is as important for academic performance as cognitive ability [17]. In the workplace, conscientiousness predicts important outcomes like job performance and job satisfaction [18–21]. Other personality factors such as Agreeableness and Neuroticism, can predict counterproductive work behavior and performance in teams [22]. As such, a single SJT measure of personality can be used to predict many different relevant outcomes, thus saving time and resources compared to developing specific SJT batteries for different outcomes. In addition, the rank-order stability of personality is high compared to, for instance, job knowledge [23], and as such, personality SJTs may also be better suited to predict future behavior. Developing a comprehensive SJT measure of personality thus enables researchers and practitioners to subsequently match relevant traits to outcomes and achieve reasonable predictive validity with a relatively small amount of work [2].

There are also several potential advantages of using SJTs to measure personality constructs as compared to using traditional self-report measures. First, SJTs are demonstrably less prone to faking than traditional self-report measures [24–26]. SJT scores showed much smaller mean level differences between faking and regular instruction conditions than self-report measures. The extent to which participants were able to increase their SJT scores seemed only to be related to cognitive ability, whereas faking in a self-report context is influenced by a magnitude of factors, for instance other personality traits [26]. SJTs also display less adverse impact than self-report Likert type scales for subgroups such as gender and ethnicity [5,27,28]. In addition, SJTs can also reflect subtler judgment processes by relating specific behaviors to situations, and may thus enhance the measurement of personality constructs. In a training context, SJTs can also be easily applied as formative assessments by elaborating the purposefulness or consequences of each response option in the respective context.

Nonetheless, we also want to point out that SJT measures of personality are not yet well established. While Mussel and colleagues [13] developed SJT measures of the NEO-PI-R facets [29] that correlate considerably with the original NEO-PI-R scales [30], ranging from a correlation of .41 for the Agreeableness facet Compliance to .70 for the Openness facet Openness for Ideas, Lievens and Motowidlo [31] suggested that the correlation between SJTs and personality can be attributed to a related, but distinct construct, namely the knowledge about the usefulness of having high or low levels of a given personality trait. This type of knowledge, referred to as implicit trait policies [32], represents the knowledge about the effectiveness of specific personality-related behaviors in the situations presented by SJTs. The theory of implicit trait policies argues that people with high levels on a trait also know about the utility of the trait related behaviors in specific situations. As such, these people will also be more likely to endorse these behaviors in SJT-type assessments. The small to moderate correlations found between many SJTs and personality traits [2,33,34] can thus be attributed to this implicit knowledge about the effectiveness of the traits and related behaviors. While we apply a construct-based approach in this study to develop SJT measures of personality, low correlations between the SJTs and classical personality measures may be indicative that the SJTs measure implicit trait policies instead.

### Facets versus broad domains of personality

Broad trait domains such as personality factors should be seen as overarching second-order factors on top of more specific first-order factors–often labeled facets [29,35]. For example, the Big Five Factor Conscientiousness can encompass facets such as Dependability, Dutifulness, or Discipline. Measuring the specific underlying facets can be even more advantageous, for several reasons. First, as the content area of a facet (e.g., Dependability) is more specific than a domain (e.g., Conscientiousness), measurements of facets can capture elements of personality with a higher fidelity than scales based on the broad domains alone [36]. This also makes tests of personality facets easier to develop, as construct definitions are more specific than for broad domains. In addition, the more specific facet measures have shown to have higher test criterion evidence than the broad trait measures. Facet measures can show stronger relations to outcomes than general trait domains by capturing relevant aspects more precisely [9].

Dependability is a core facet of Conscientiousness and one of the best predictors of overall job performance of the Conscientiousness facets [19]. A person with high Dependability is reliable, responsible, fulfills obligations and respects authority. Dependability has been rated as the most valued work style or attribute by employers in the evaluation of the United States Department of Labor’s Occupational Information Network [21]. Dependability is ranked in the top 3 valued traits for 19 out of 23 job families covering approximately 1,102 occupations. These data provide support for the potential value of developing a Dependability SJT measure.

### Current investigation

The main goal of this investigation is to further examine the validity of newly developed SJT measures of personality constructs in two studies. This was achieved by developing innovative SJTs following recommended best practices in SJT construction and conducting psychometric studies designed to evaluate the reliability and validity of these measures. We will examine whether the new construct-based personality SJTs are reliable and valid measures of the personality construct Dependability. We will also examine the impact of different scoring procedures on the psychometric quality of these types of SJTs. After construct validity has been established, we will examine the criterion-related validity of the new type of construct-based SJT as compared to typical self-report measures of personality.

## Study 1

The main aim of Study 1 was to examine the psychometric quality of newly-developed construct-based SJTs. SJTs were developed to measure Dependability, a core facet of Conscientiousness. Two scoring procedures were compared, one based on expert ratings and one based on the sample distribution (i.e., consensus scoring). We examined the impact of the scoring procedure on construct validity evidence by relating SJT scores to other personality assessments, such as the Big Five Inventory [37], and on structural validity evidence through a one-factor confirmatory factor analysis (CFA) of the 18 SJT items (as we expected all 18 SJTs to measure a common Dependability factor). We then compared the Dependability SJT scores with scores derived from alternative measurement methods of Dependability (a self-report rating scale and a self-report biographical data questionnaire). To further examine whether SJTs capture individual differences in personality, we specified a multi-method CFA model accounting for common trait and specific assessment method variance across the three measures of Dependability. Under the assumption that the SJTs do indeed measure personality instead of implicit trait policies, we predicted the following results:

- The SJTs will yield acceptable model fit and reliability for the one factor model encompassing all 18 SJTs.
- The SJTs will correlate moderately with the Dependability self-report and biographical data questionnaires.
- The SJTs will correlate moderately the BFI measure of Conscientiousness.
- The SJTs will not correlate with the other Big Five factor scores.

### Method

The study conforms to Standard 9 of the American Psychological Association’s Ethical Principles of Psychologist and Code of Conduct. The sample consists of adults that participated voluntarily in this study. Consent was informed. At the start of the study, participants were informed that they could abort the survey at any time and still receive full compensation. By beginning the study, consent was given. No personal identifiers (e.g., Social Security Number) were collected.

#### Participants

Participants were 600 Amazon Mechanical Turk (AMT) workers who were residents of the United States. AMT has the benefit of providing fast recruitment of samples that are demographically more diverse than typical college or internet samples [38,39]. The quality of the data collected in AMT is reported to be at least as reliable as other data collection methods [38–40]. The majority of AMT workers also seem to participate for intrinsic reasons (e.g., entertainment) and may be more motivated to complete the tasks given. From our initial sample of 600 participants, we excluded 54 people (9%) who either did not complete the study or failed to provide correct answers to at least 3 out of 5 instructed-response questions designed to identify random or other forms of inattentive responding [41]. The mean age of the remaining 546 cases was 34.5 years (*SD* = 10.2). In this sample 293 participants were female. Half of the sample held at least a bachelor’s degree. Participants were given $4 for their participation in the 30-minute survey, which is much higher than the median AMT compensation rate of $1.38 per hour [42].

#### Measures

Dependability SJTs. Semi-structured interviews were held with five individuals in full-time work (three males and two females), all but one of whom had obtained a university degree. The researcher took notes as the interviews progressed. The standard question prompt was varied to include content phrases indicating high and low levels of dependability: “Tell me about a time when you or a colleague of yours has <*insert term from construct definition below>*. What was the situation? What happened?” High dependability phrases included: been reliable, been responsible, been dependable, been industrious/hard-working; been efficient; been punctual; been consistent; shown a strong work ethic; been well-prepared; made and stuck to their plans. Low dependability phrases included: been unreliable, been lazy, been frivolous, wasted time; shirked their duties; not followed through on plans, left things unfinished. Follow-up questions asked for clarification of the behaviors, with the standard prompt “what did they do?” and requests for further detail regarding the context of the behavior if this was unclear. The high versus low descriptors were drawn from the O*Net descriptions of Dependability [43], and edited for clarity and ease of understanding. Based on these situation descriptors, three to five sentence descriptions of situations were created, along with five possible responses that intentionally varied from low to high dependability.

The situations were not contextualized to any specific profession, but reflected general work situations instead, such that the instrument would be relevant to a broad range of occupations, as well as work-readiness assessments for people entering the job market for the first time. As such, these situations have little reliance on occupational knowledge.

The behavioral instruction for the SJTs read, “How likely are you to respond with each of following actions?” Respondents answered to each response option on a 5-point Likert scale ranging from “Very Unlikely” to “Very Likely” An example of the resulting SJTs is presented below:

“You are asked to deliver a critical report to your supervisor by close of business today. At your company, reports such as this one are supposed to be prepared according to specific procedures and guidelines. If you follow all the steps in the order suggested, however, you will not meet the deadline.”

How likely are you to respond with each of following actions?

1. Keep working on the report, following all procedures and guidelines, and give your supervisor whatever you have completed by the end of the day.
2. Follow the procedures and guidelines and work into the night so you can deliver the completed report by start of business tomorrow.
3. Tell your supervisor that you cannot complete the report by close of business today.
4. Ignore the procedures and guidelines and do only the most essential parts of the report to meet the deadline.
5. Ignore the procedures and guidelines, but take as much time as you need to do the job.

We included a number of additional personality measures to examine the validity of our SJTs. In addition to including a well-established measure of the Big Five, we developed self-report and biographical data measures of Dependability to examine the construct validity of the SJTs with different assessment methods of the same construct in a multi-method design.

Big Five Inventory. The Big Five Inventory (BFI) [37] is a 44 item measurement of the Big Five trait domains. Each item (e.g. “I see myself as someone who does a thorough job”) is measured on a five-point Likert scale ranging from “Strongly Disagree” to “Strongly Agree”.

Dependability self-reports. We developed 30 self-report items measuring dependability (e.g., “I start tasks right away”, “I leave things unfinished”) based on the O*Net descriptions of Dependability [43]. The items were developed to capture all aspects listed in the definition of Dependability, thus providing a broad construct coverage. Half of the items were reverse coded. Each item was measured by a six-point Likert scale ranging from “Strongly Disagree” to “Strongly Agree”.

Dependability biographical data measure. We additionally developed 18 biographical data (checklist) items assessing past behavior (e.g., “Taken more than one day to return a phone call”, “Given someone useful advice”) with the instruction “To which extent have you engaged in each of the following behaviors in the last year?” Again, we tried to select behaviors that allowed us to capture all aspects of the Dependability definition. Each biodata item was answered on a six-point Likert scale ranging from “Never” to “Always”.

#### SJT scoring procedures

Expert scoring. We asked four subject matter experts from industrial-organizational and personality psychology to rate each response option on the extent to which it was representative of Dependability, on a five-point Likert scale from “very undependable” to “very dependable”. Across all 89 response options (one was excluded for being a data-check item) the overall mean of the expert ratings was 2.99 (*SD* = 1.31; on a scale from 1 to 5), which suggests that the desirability of responses was evenly balanced across all SJTs. The Intra-Class Correlation between the four raters was .66.

To account for varying response styles (e.g., some people using the extreme ends of the scales, some using only one end of the scale), we intra-individually *z*-standardized raw scores across all SJT responses (i.e., a person’s ratings were converted to *z*-scores, so that each person had a mean of 0 and a standard deviation of 1 across all responses). The expert rating profile was also *z*-standardized. We then computed the absolute difference between the respondents’ and expert standardized scores on every response option. Scores were added up for every SJT. As higher scores reflect a higher deviation from the expert profile, scores were subsequently reversed by subtracting them from 0.

Consensus scoring. We computed the sample proportions in each response option and weighted the respondents’ selection based on these proportions. For example, if 32% of the sample chose “very likely to do” on a response option, this option will be scored with 0.32. Scores across response options were added up for every SJT. A simplified example both SJT scoring procedures can be found under https://osf.io/uacb6/.

### Results

#### Dependability self-report and biodata scales

We evaluated each of the newly developed scales by testing the model structure with CFA. We specified one-factor models for each scale and estimated the models using the *MLR* estimation in Mplus 7 [44]. The 30-item self-report dependability yielded insufficient model fit (χ^2^ = 1,868; *df* = 405; CFI = .79; RMSEA = .08; SRMR = .06) [45]. However, the source of model misfit was unclear, as all items yielded sufficient loadings. One possibility might be the large number of indicators, which is often a problem for self-report scales [46]. We thus used the item selection algorithm Ant Colony Optimization [47,48] to identify the 18 items that would optimize the CFI and RMSEA value of the model. The resulting 18-item model fitted the data well (χ^2^ = 322; *df* = 135; CFI = .94; RMSEA = .05; SRMR = .04) and yielded good factor saturation (McDonald’s *ω* = .93). The one-factor 18-item biodata model yielded bad model fit (χ^2^ = 667; *df* = 135; CFI = .70; RMSEA = .09; SRMR = .09). Five items yielded factor loading close to zero, suggesting that these items do not measure Dependability. After removing these items, the 13-item model yielded acceptable model fit (χ^2^ = 185; *df* = 65; CFI = .90; RMSEA = .06; SRMR = .05) and factor saturation (*ω* = .85). We thus used the shortened scales for the subsequent analysis. Factor loadings for the models can be found in the online repository under https://osf.io/uacb6/.

#### SJT scoring

The Expert based and Consensus SJTs scores correlated around *r* = .80 (*p* < .01). However, model fit of the unidimensional CFA models differed strongly between the scores. We estimated one-factor models for both scoring procedures with *MLR* estimation. The Expert scores resulted in good model fit (χ^2^ = 189; *df* = 135; CFI = .95; RMSEA = .03; SRMR = .04, *ω* = .78), whereas the Consensus scores showed poor fit to the data (χ^2^ = 666; *df* = 135; CFI = .67; RMSEA = .09; SRMR = .08, *ω* = .80).

#### Correlation with personality scales

Table 1 shows the correlations between the SJT scores and personality self-report measures. Consensus-based SJT scores yielded only small correlations with the self-report and biographical data measures of Dependability. The correlations with Conscientiousness as measured by the BFI was not significant. The Expert score showed moderate correlations with the other measures of Dependability (self-report: *r* = .47; *p* < .01; biodata: *r* = .29; *p* < .01) and the Conscientiousness measure (*r* = .33; *p* < .01). As expected, correlations with the other measures of Dependability are higher than correlations with the broad Conscientiousness factor measured by the BFI. While the Expert-scored SJTs correlate highest with the Conscientiousness factor in the BFI, the correlation with Agreeableness (*r* = .30; *p* < .01) is also substantial and close in magnitude to the correlation with Conscientiousness. This finding can be attributed to the social context of the SJTs, in which agreeable behaviors (e.g., helping others) are also indicative of Dependability. Note that correlations between self-report measures of Agreeableness and Conscientiousness (*r* = .42; *p* < .01) or Dependability (*r* = .45; *p* < .01) are also very high in this sample and might indicate social desirability effects.

**Table 1:** Correlations of the dependability scales with self-report measures of personality.

	*M*	*SD*	*α*	Co-SJT	Ex-SJT	D. SR	D. BD	C	A	N	O	E
Co-SJT	1.55	0.16	.78									
Ex-SJT	-3.67	0.70	.78	.80								
D. SR	4.65	0.77	.92	.19**	.46**							
D. BD	5.03	0.59	.85	.21**	.29**	.56**						
C	3.97	0.70	.89	.08	.33**	.84**	.50**					
A	3.85	0.66	.83	.10*	.30**	.43**	.21**	.42**				
N	2.59	0.93	.91	.05	-.10*	-.45**	-.32**	-.52**	-.43**			
O	3.75	0.68	.87	.10*	.18**	.19**	.10*	.27**	.21**	-.23**		
E	3.00	0.93	.91	-.13**	.00	.36**	.13**	.39**	.37**	-.51**	.35**	

*Note*. Co = Consensus scoring; Ex = Expert scoring; SJT = Situational Judgment Test; D. = Dependability; SR = Self-Report; BD = Biographical data; C = BFI Conscientiousness; A = BFI Agreeableness; N = BFI Neuroticism; O = BFI Openness; E = BFI Extraversion; *α* = Cronbach’s alpha

* *p* < .05

** *p* < .01

#### Multi-method model

**Fig 1:**
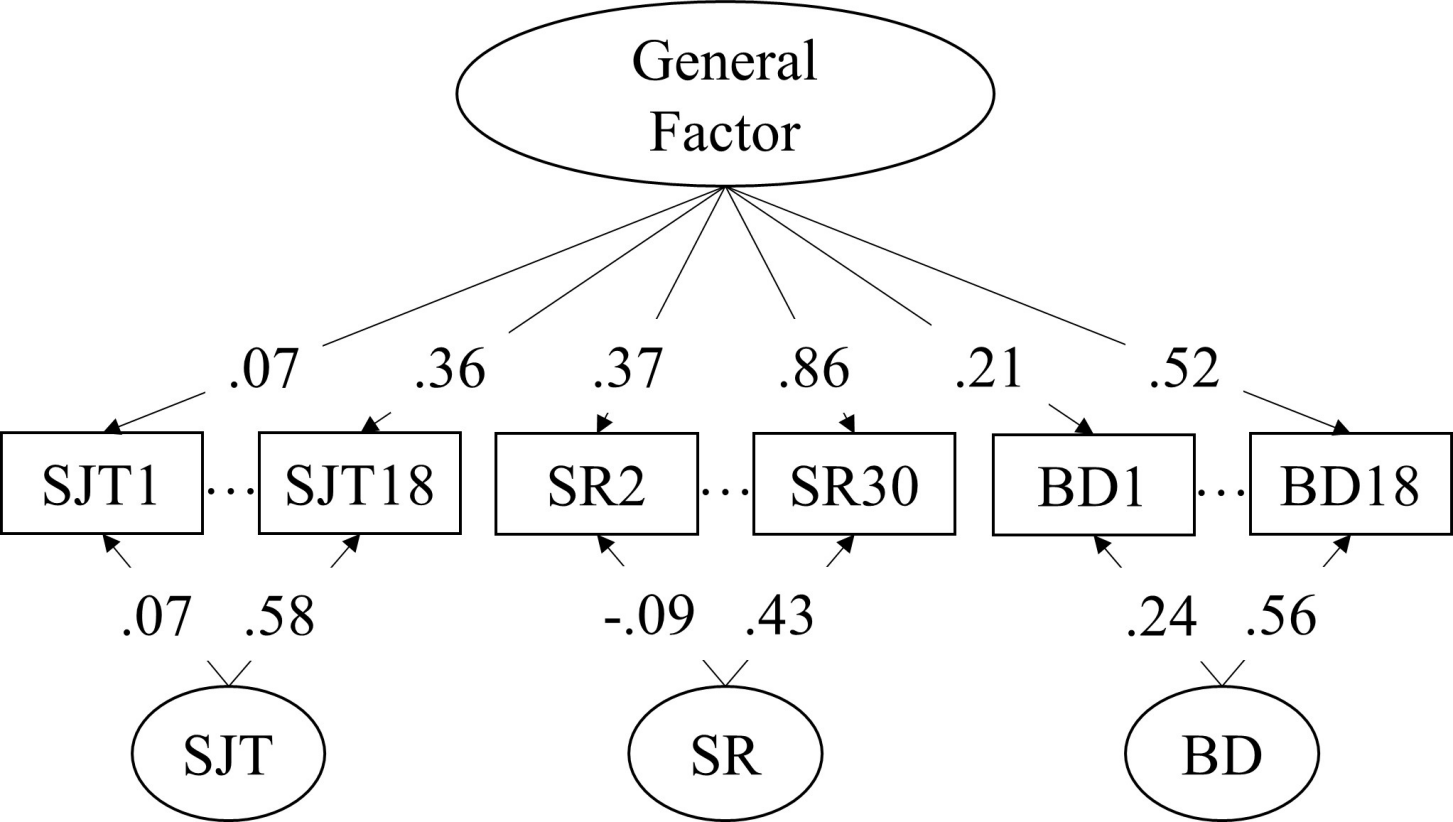
Multi-method bi-factor model of dependability. SR = self-report; BD = biodata. The loadings presented represent the standardized loading range of the corresponding scales. Negative loadings on the SR and BD factors result from response effects (e.g., acquiescence) on negatively coded items. Model fit: CFI = .90; RMSEA = .04; SRMR = .05.

To examine the unique proportion of variance in the SJTs compared to the other measures of Dependability, we estimated a bi-factor model on all three Dependability measures with a general Dependability factor and uncorrelated specific nested factors for SJTs, self-report and biodata measures (see Fig 1). The nested factors are intended to capture the unique method variance of each test format. However, note that the nested factors might also include differences in the construct coverage (we tried to minimize this by developing all three measures based on the O*Net definition of Dependability).

Goodness-of-Fit indices of the model with *MLR* estimation were acceptable (χ^2^ = 1,802; *df* = 1,078; CFI = .90; RMSEA = .04; SRMR = .05). The self-report items yield the highest loadings on the general Dependability factor (average λ = .70; see https://osf.io/uacb6/ for full loading structure) as well as lowest specific factor loadings (average λ = .19). In contrast, the loadings of the SJT items were stronger on the specific factor (average λ = .34) than on the general factor (average λ = .21), suggesting that a large portion of the SJT variance captures unrelated individual differences. Biodata items loaded slightly higher on the general factor (average λ = .36) than on the specific factor (average λ = .26). Table 2 shows the correlation between the four factors and the BFI scores. The overall Dependability factor correlated very highly (*r* = .82; *p* < .01) with BFI-C, supporting the notion that the three scales measure a central aspect of the trait. Correlations between BFI-C and the SJT and biodata factors were close to zero. The somewhat larger relationship between the self-report nested factor and BFI-C can be attributed to the method-effect of self-report items (correlation with the self-report factor: *r* = .44; *p* < .01), which are not present when using the SJT method (correlation with the SJT factor: *r* = -.06; *p* > .05). Correlations of the Dependability factor with the BFI-A scores were moderate (*r* = .41; *p* < .01), showing that the correlation between the Dependability scales and Agreeableness is mostly driven by similarities between the constructs or potential social desirability effects. The social aspect of the SJT situations does not seem to contribute to the zero-order correlation between SJTs and BFI-A shown in Table 1.

**Table 2:** Bi-factor model correlations with the bfi personality scores.

Factor	C	A	N	O	E
General	.78**	.39**	-.42**	.10	.33**
SJT (S)	-.06	.07	.15*	.15*	-.23**
SR (S)	.44**	.27**	-.29	.39**	.22
BD (S)	.07	-.02	-.12	.09	-.09

*Note*. SJT = Situational Judgment Test; SR = self-report; BD = Biodata; (S) = specific factor; C = BFI Conscientiousness; A = BFI Agreeableness; N = BFI Neuroticism; O = BFI Openness; E = BFI Extraversion

* *p* < .05

** *p* < .01

### Discussion

The CFA findings support the unidimensionality of the 18 SJT scores. The SJTs in this study were moderately related to self-report and behavioral frequency checklist measures of Dependability and Conscientiousness. While correlations with the other Dependability measures were similar to findings by Mussel and colleagues [13], the relatively low correlation with Conscientiousness and the low Dependability factor loadings in the multi-method model suggest that only a small to moderate proportion of the SJT variance is related to personality. There are several potential explanations for this effect: One explanation for this finding could be that the SJTs capture implicit trait policies [32] instead of the personality traits directly. The correlation between the SJTs and self-report measures of Dependability or Conscientiousness is also arguably reduced due to the scoring procedure applied. As we intra-individually z-standardized SJT responses and compared them to the expert profile, scale usage effects (e.g., acquiescence) are eliminated, whereas these might have artificially increased the correlation between the self-report scales. In addition, SJTs are also less prone to faking and social desirability effects compared to the traditional measures of personality. This might have further reduced the correlation between the different assessment methods. These explanations are also supported by the relatively high correlations between the different BFI scales. Surprisingly, the SJT correlations with self-reported Agreeableness were nearly as high as the correlation with Conscientiousness. However, as the multi-method model showed, this correlation can be attributed to the relation between Dependability and Agreeableness instead of specific SJT variance. The construct definition of Dependability also encompasses fulfilling obligations and respecting authority, which seem to be related to the Agreeableness facets Cooperation and Compliance. In comparison, the self-report scales of Conscientiousness correlated more highly with Agreeableness than the SJT scale (.42-.43 vs. .30), also suggesting a reduced impact of scale usage and social desirability in the SJTs.

The Consensus scoring procedure performed substantially worse than the Expert scored SJTs. Model fit was insufficient for the Consensus-based scores, and correlations with other measures of Dependability and Conscientiousness were substantially lower. Consensus scoring may be problematic in this context for a number of reasons. In a maximal performance setting, the scoring procedure is problematic for SJTs with higher difficulty, as they may not be correctly solved by a large proportion of the sample. The difficulty of SJTs can be artificially reduced or distorted, as responses are scored based on their perceived effectiveness by a sample with usually less insight than experts. When measuring typical behavior, this scoring procedure will result in more heterogeneous scores, as the responses do not converge towards an “optimal” or “correct” response. In addition, the Consensus scoring procedure will assign the highest score to participants that respond similarly to the rest of the sample, thus arguably favoring responses in the middle of the scale. In contrast, the Expert scoring is independent of scale usage effects because of the *z*-standardization and transforms the raw SJT responses into a difference metric based on a common expert profile. The resulting scores are thus much more homogenous than the Consensus scores.

## Study 2

The goal of the second study is to replicate the findings from Study 1 and gather additional validity evidence for the newly developed SJTs by examining the criterion-related validity in a sample working in sales. Work-related outcomes were measured by assessing job performance–task performance (the percentage of sales objective and income goal reached last year) and counterproductive workplace behavior [49]–as well as variables that indicate workplace wellbeing (job satisfaction and turnover intentions).

In addition to examining construct validity in the same manner as in Study 1, we will examine whether the Dependability SJTs are capable of predicting work-related outcomes. Based on previous findings on the relationship between Conscientiousness and general job performance [18,19] or sales performance [18,50] we expect the Dependability SJTs as a measure of a core facet of Conscientiousness to correlate positively with measures of job performance. We also expect the SJTs to be positively related to work satisfaction [51] and negatively to counterproductive workplace behavior and turnover intentions [16,51–53]. In addition, we expect the SJTs to provide incremental validity in predicting performance beyond classical self-report measurements of personality [54,55]. In addition to the construct validity hypotheses proposed in the previous study we predict the following:

V The SJT method will predict task performance measures incrementally beyond other measures of Dependability.
VI The SJTs will predict counterproductive workplace behavior incrementally beyond other measures of Dependability.
VII The SJTs will predict job satisfaction and turnover intentions incrementally beyond self-report measures of Dependability.

### Method

#### Participants

A total of 402 participants were recruited on Amazon Mechanical Turk. The explanation of the study specifically stated that only people currently working as sales professionals should participate. Fifteen cases (3.7%) were discarded based on failing at least 3 out of 4 questions designed to identify random or inattentive response patterns. The mean age of the remaining 387 participants was 32.6 years (*SD* = 8.6). Out of the sample 47% had at least a bachelor’s degree. The work field with the highest representation was “Grocery and related products” with 22.5% of the sample. The majority of participants (68%) reported an income of less than $60K a year (17% below $20K; 24% between $20K and $40K; 27% between $40K and $60K). Income levels are thus lower than for the general US population, but similar to previous findings on the income of AMT workers [40]. Participants were paid $5 for their participation.

#### Measures

In line with the previous study, this study included the Dependability SJTs, the BFI [37], as well as the shortened 18-item self-report and 13-item biographical data measures of Dependability. We additionally included the following outcome measures:

Counterproductive Workplace Behavior. Counterproductive Workplace Behavior **(**CWB) was measured with 19 items capturing the two aspects of organizational and interpersonal counterproductive workplace behavior. Organizational CWB measures negative behaviors towards the organization (e.g., stealing office supplies). Interpersonal CWB captures negative behavior towards coworkers (e.g., bullying). Respondents were asked to report how often they engaged in counterproductive workplace behaviors during the last year (e.g., “Come in late to work without permission”) on a seven-point Likert scale ranging from “Never” to “Daily”.

Sales outcomes. We derived outcome questions based on an interview with a sales director in a company with 70 employees. We included single response self-report questions intended to measure sales performance. Respondents were asked whether they received a raise or promotion in the last two years, what percentage of their sales quota they reached last year on a scale from “Below 50%” to “Above 100%” (in increments of 10%), and the percentage of their personal income goal they reached on a scale from “Below 50%” to “Above 100%” (in increments of 25%).

Job satisfaction and turnover intentions. Participants were also asked about their overall job satisfaction on a five-point Likert-scale ranging from “Very dissatisfied” to “Very satisfied”. Turnover intentions were assessed with the two self-report questions “How frequently do you consider leaving your current position?” and “How frequently do you consider leaving the profession?” using a five-point Likert-scale ranging from “Very infrequently” to “Very frequently”.

### Results

#### Construct validity evidence

The 13-item biodata measure of Dependability yielded similar model fit and factor saturation as in the first study (χ^2^ = 137; *df* = 65; CFI = .92; RMSEA = .05; SRMR = .05; *ω* = .86). The self-report scale performed somewhat worse than in the previous study (χ^2^ = 344; *df* = 135; CFI = .88; RMSEA = .06; SRMR = .06) but yielded a similarly high factor saturation (*ω* = .92). Due to the poor performance of the Consensus scoring procedures we only applied Expert scoring to the SJTs in this study. Similar to the previous study, the Expert scoring yielded good model fit (χ^2^ = 192; *df* = 135; CFI = .94; SRMR = .04; RMSEA = .04) and factor saturation (*ω* = .83). Table 3 shows the correlations between the different measures of Dependability and the Big Five. Correlations between the SJTs and other Dependability measures were higher (all *p* < .01) in this sample (self-report: *r* = .57; biographical data: *r* = .60) than in Study 1. The correlations with Conscientiousness (*r* = .44) and Agreeableness (*r* = .40) were moderate. Note that the BFI Conscientiousness and Agreeableness scales were highly correlated (*r* = .52).

**Table 3:** Correlations of the dependability and BFI scales.

	*M*	*SD*	*α*	D. SJT	D. SR	D. BD	C	A	N	O	E
D. SJT	-4.01	0.84	.83								
D. SR	3.92	0.61	.91	.57**							
D. BD	4.90	0.66	.85	.43**	.61**						
C	4.07	0.67	.88	.44**	.84**	.58**					
A	3.90	0.70	.84	.40**	.56**	.37**	.52**				
N	2.35	0.87	.86	-.14**	-.43**	-.42**	-.53**	-.40**			
O	3.69	0.65	.83	.30**	.35**	.18**	.36**	.26**	-.16**		
E	3.24	0.89	.89	-.01	.24**	.16**	.33**	.20**	-.50**	.16**	

*Note*. D. = Dependability; SR = Self-Report; BD = Biographical data; C = BFI Conscientiousness; A = BFI Agreeableness; N = BFI Neuroticism; O = BFI Openness; E = BFI Extraversion

** *p* < .01

#### Criterion-related validity evidence

Correlations of the Dependability and BFI scales with the assessed outcomes are presented in Table 4. As expected, all three Dependability scales and BFI Conscientiousness showed moderate to high negative correlations with counterproductive workplace behaviors. The scales also yielded small positive correlations with job satisfaction and percentage of sales and income goals reached, as well as small negative correlations with turnover intentions. However, the SJTs correlated lower with the outcomes than the self-report scales. The only exception was the percentage of the income goal reached (SJTs: *r* = .21, *p* < .01), which showed the strongest correlation with the SJTs.

**Table 4:** Correlations between personality and work-related outcomes.

	CWB-I	CWB-O	% sales objective	% income goal	Job satisfaction	Turnover intentions
*M*	1.50	1.84	6.10	4.33	3.95	2.39
*SD*	0.81	0.90	1.79	0.80	0.81	1.19
*α*	.87	.88				
D. SJT	-.31**	-.34**	.14**	.21**	.03	-.05
D. SR	-.36**	-.53**	.14**	.14*	.24**	-.22**
D. BD	-.51**	-.65**	.17**	.14*	.18**	-.15**
BFI C	-.33**	-.52**	.15**	.16**	.29**	-.24**
BFI A	-.42**	-.37**	.08	-.02	.26**	-.17**
BFI N	.22**	.33**	-.21**	-.13	-.36**	.29**
BFI O	-.09	-.07	.13*	.10	.17**	-.01
BFI E	.04	-.13*	.11*	.19*	.30**	-.28**

*Note*. D. = Dependability; SR = self-report; BD = biodata; C = Conscientiousness; A = Agreeableness; N = Neuroticism; O = Openness; E = Extraversion; CWB = counterproductive workplace behavior (I = Interpersonal; O = Organizational); % sales objective = percentage of sales objective reached last year; % income goal = percentage of income goal reached last year.

* *p* < .05

** *p* < .01

To account for specific method variance (again note that this might also include differences in the construct coverage), we divided the overall variance of the Dependability scales into general (i.e., Dependability) and specific (i.e., SJTs, self-report, and biodata) variance by again applying the bi-factor model with a common Dependability factor and orthogonal nested specific factors (see Fig 1). The model again yielded acceptable fit (χ^2^ = 1,623; *df* = 1,078; CFI = .90; RMSEA = .04; SRMR = .05). While SJTs still yielded the highest method and lowest trait factor loadings (average specific factor λ = .35; average general factor λ = .30; see https://osf.io/uacb6/ for full loading pattern), the discrepancy was not as large as in the previous study. The loadings of the self-report and biodata items were similar to the previous study (general factor: average self-report λ = .67; average biodata λ = .41; specific factors: average self-report λ = .11; average biodata λ = .21). The correlations with the BFI scores were also similar to the previous study, most notable was the high correlation between the general factor and BFI Conscientiousness (*r* = .87; *p* < .01). The generalizability of the model across samples is thus supported. The correlations between the factors and outcomes are presented in Table 5. As expected, the overall Dependability factor was related to lower counterproductive workplace behavior and turnover intentions, as well as higher job satisfaction and percentage of sales objectives and income goals reached. The specific SJT variance was positively related (*r* = .20; *p* < .05) to the income goal reached. Surprisingly, a higher SJT score also seemed to result in lower job satisfaction (*r* = -.18; *p* < .01), as well as higher turnover intentions (*r* = .16; *p* < .01) after accounting for the common variance across the three Dependability measures. This might indicate that participants with higher scores in such low fidelity work situations also have a higher tendency to leave their current position, possibly because they feel they deserve better employment opportunities. It is also noteworthy that the biodata items seem to be particularly well suited to predict counterproductive workplace behavior (Interpersonal: *r* = -.38; *p* < .01; Organizational: *r* = -.40; *p* < .01). This high correlation can be attributed to both scales referring to specific behaviors in the last year and the biodata items showing high similarities to CWB items (e.g., “were late to a meeting”, “criticized someone in front of others”).

**Table 5:** Bi-factor model correlations with work-related outcomes.

Factor	CWB Interpersonal	CWB Organizational	% sales objective	% income goal	Job Satisfaction	Turnover intentions
General	-.38**	-.58**	.13*	.11	.25**	-.23**
SJT (S)	-.08	.03	.08	.20*	-.18**	.16**
SR (S)	.02	.21**	.08	.02	.10	.09
BD (S)	-.38**	-.40**	.11	.03	.06	-.01

*Note*. SJT = Situational Judgment Test; SR = self-report; BD = Biodata; (S) = specific factor; CWB = counterproductive workplace behavior; % objective = percentage of sales objective reached last year; % income goal = percentage of income goal reached last year. Values in the *Average trait/method factor loading* column represent the absolute mean loading of the items (all items for Dependability) on the trait (i.e. Dependability) and corresponding method factor

* *p* < .05

** *p* < .01.

### Discussion

The second study yielded larger correlations between the SJTs and related self-report measures than the first study, as well as higher trait factor loadings in the multi-method model. Similar to the previous study, the SJTs correlate most strongly with other Dependability measures, followed by Conscientiousness and Agreeableness. The expert-based SJT scores resulted in good model fit and substantial correlations with the outcome measures. As expected, the Dependability SJTs were negatively related to CWB, as well as positively to the task performance. Contrary to expectations, these correlations were lower than the correlations of the related self-report scales with the outcomes—the exception being the percentage of the income goal reached. Arguably, the self-reported outcomes might have benefitted self-report scales in this regard and more objective outcome measures are desirable for future studies.

## General discussion

The goal of this paper was to examine the validity of a new set of construct-based personality SJTs. We examined the construct and criterion-related validity of the newly developed measures across two studies covering a general and a sales-specific sample.

The Dependability SJTs correlated moderately to highly with self-report and behavioral frequency checklist measures of Dependability. The correlations were relatively large given the differences between the measurement methods and scoring procedures (i.e., intra-individually *z*-standardizing responses and comparing them to an expert profile). The findings reported here surpass the correlations generally reported on personality measures and SJTs in meta-analyses [2] and were of similar magnitude as correlations reported for other personality SJTs [13]. While this supports the validity of the newly developed SJTs as measurements of Dependability and provides evidence in favor of SJTs as measures of personality, the multi-method model showed that the SJTs also capture a similarly high (study 2) or even larger proportion (study 1) of method specific variance. There are several potential explanations for this finding: As suggested by Lievens and Motowidlo [31], the SJTs might measure implicit trait policies instead of personality traits directly [32], and the specific SJT variance may represent the knowledge component. Alternatively, the general factor might also capture scale usage and social desirability variance from the self-report and biodata measures. As the SJTs and corresponding scoring procedure were intended to eliminate such effects, the SJT loadings on the general factor might have been reduced. To evaluate these perspectives, it is desirable to include independent measures of implicit trait policies and social desirability in future studies.

The criterion-related validity findings for the newly developed set of SJTs are also noteworthy. The newly developed SJTs have shown the same relationship with job performance reported in meta-analyses of Conscientiousness [18–21,56] or SJTs in general [33]. Arguably, the correlation was reduced by the low income levels of the participants in the second study and the large proportion of sales workers in groceries or retail, where the behaviors assessed in the SJTs may be of smaller relevance to work performance. This might have also resulted in the positive correlation found between the SJT factor and turnover intentions (or negative correlations with job satisfaction respectively; see Table 5). The low income and arguably low job status of the participants (e.g., working in retail) given the otherwise higher education (i.e., the majority of participants held at least a bachelor’s degree) might have resulted in a low job satisfaction and high turnover intentions. While Dependability seemed to predict these two aspects of work satisfaction as predicted, participants that scored higher on the SJTs might seem to feel overqualified for their current employment.

The validity of the final SJT scores is not only dependent on content and design, but also on the scoring procedure applied. Consensus scoring was inferior to Expert-based scoring in regard to validity. While not presented in this article, we also examined the Consensus scoring in the second sample and found similar results as in the first study and non-significant correlations with the outcome measures. In addition to some of the flaws of a sample distribution based approach discussed previously (i.e., skewed distribution, distorted item difficulty), the Likert-scale response format can have also affected the Consensus scoring negatively, due to scale usage and acquiescence effects. The Expert scoring procedure explicitly aimed at eliminating the effect of such response tendencies and has been demonstrated to yield satisfactory results. More relevant in regard to the poor performance of the Consensus scores might have been the instruction used. Since respondents were asked to provide their likelihood of demonstrating the behaviors, responses do not gravitate towards the “correct” response, but instead represent the Dependability distribution of the sample. As such, respondents with more typical responses (i.e., show medium levels of Dependability), will reach higher scores. Independent of this, using an expert profile as a gold standard reduces issues of sample specificity and makes the scoring procedures more comprehensible for practitioners and participants.

Given the somewhat lower criterion related validity of the SJTs (compared to the self-report measures), what are the benefits of developing SJT measures of personality? First, we want to point out that most of the outcome measures were assessed via Likert scale items (e.g., counterproductive workplace behavior, job satisfaction, turnover intentions). As such, it is possible that the correlations between the self-report scales and these outcomes are artificially increased due to scale usage effects or social desirability (note that the SJTs preformed similarly or better than self-reports when predicting more objective outcome measures, such as the percentage of sales or income goal reached). One advantage of the SJT method is that the presentation of dilemmas and the expert scoring procedure will eliminate such effects, thus providing a more truthful measure of the underlying traits. This is in line with comparisons of faking between SJTs and self-report scales [24–26]. As mentioned earlier, SJTs also show less adverse impact on ethnicity or gender than classical self-reports [5,27,28]. While SJTs may be cognitively more demanding than Likert-scale assessments, participants in our study also reported generally higher engagement on this item type, thus potentially reducing fatigue or careless responding.

While SJTs have typically been used as selection tools, this method can also be used as a formative assessment. In personality research, this is particularly interesting, as recent studies have shown that the personality traits can be changed with specifically targeted interventions [57–59]. By changing relevant behaviors or habits, long-term development of the underlying traits can be achieved. SJTs can be used in this context to educate participants on how different behaviors shape consequences and what the ideal or desired responses are on every response option. Justifications of the expert ratings can be presented to explain why each behavior demonstrated a certain level of effectiveness or personality trait. To do so, subject matter experts should also be asked to provide explanations for their rating of response options. These justifications can then further help educate test-takers as to which behaviors are more effective or desirable.

### Limitations and future directions

In this article we presented and examined only one of several possible types of construct-based personality SJT. Future studies can, for example, examine whether all Big Five factors can be measured with similar validity. In addition, the impact of the SJT design on the validity should be examined in future studies. How will instruction type, response format, and scoring procedure influence the validity of personality SJTs? A noteworthy study where the influence of instructions on otherwise unchanged SJTs was conducted by McDaniel and colleagues [33], but such studies are few and have not yet been conducted for construct-based SJT measures of personality. We developed SJTs with work-related situations to potentially increase the correlation with work-related outcome measures. As such, the SJTs presented here are only applicable to working respondents, and need to be generalized more for non-working samples [13].

The samples collected in the studies described here were recruited via Amazon Mechanical Turk. The income distribution was at the lower end of the spectrum, and a large proportion of the samples were working in retail. In regard to sales performance, future studies might want to aim at a more homogenous sample only covering one work field, in order to make outcome variables more comparable.

In this study, we were unable to reliably identify the variance components captured by the SJTs compared to self-report scales. In future studies, we suggest including measures of procedural knowledge or implicit trait policies, as well as measures of social desirability. By additionally creating SJTs measuring more than one trait, the SJT variance can be analyzed for trait, situation, method and social desirability effects using the variance decomposition approach presented by Jackson and colleagues [6,12]. Until evidence clearly suggests that SJTs are capable of capturing personality traits to a large extent, we suggest combining SJT and self-report measures of personality to increase the reliability and construct coverage of the measurement of the underlying trait.

We also want to point out that the correlations between the biodata scale and other measures of Dependability/Conscientiousness (i.e., SJTs, self-report Dependability, and BFI Conscientiousness) decreased after removal of the five items with zero-loadings. This decrease was largest for the correlation with the SJTs (a difference of .17 compared to .10 with the self-report scales). This suggests that aspects measured by the five removed biodata items were related to the variance captured by the other measures, most notably the SJTs. It might seem unusual to remove items that apparently carry some of the validity but we wanted to stress the importance of creating measures that fulfill the unidimensionality assumptions of latent trait theory [60], rather than solely relying on external correlations as an indicator for scale quality. Importantly, we apply this strategy to the biodata scale as well as to the SJTs. The later have been pointedly characterized as “psychometric alchemy” [61] because they seem to have substantial predictive but low construct validity. We hope that the construct-based approach for developing and evaluating SJTs presented here [see also 2,13] provides a blueprint for unidimensional SJT measures based on a clear construct definition. In future studies a number of unidimensional measures might be combined in order to elaborate and strengthen a nomological net.

## Conclusions

In this article we developed 18 Dependability SJTs based on a new construct-based approach to SJT development. We related these SJTs to classical measurements of personality and a broad range of job performance outcomes for sales professionals. The newly developed SJTs showed small-to-medium correlations with work-related outcomes, as well as moderate-to-high correlations with self-reported personality [13]. However, a multi-method analysis encompassing two other assessment methods of personality showed that the SJTs seem to capture a similarly large proportion of non-personality related variance. This might indicate that even construct-based SJTs measure general domain knowledge about personality traits [32] instead of personality factors directly. The negative correlation of the SJT specific variance with job satisfaction also supports the notion that the SJTs measure personality-related knowledge. Given these findings, SJTs can be used to provide formative assessments that can be used to shape personality-related behaviors and habits [57,58].
